# Preperimetric Glaucoma Prospective Study (PPGPS): Predicting Visual Field Progression With Basal Optic Nerve Head Blood Flow in Normotensive PPG Eyes

**DOI:** 10.1167/tvst.7.1.11

**Published:** 2018-01-23

**Authors:** Yukihiro Shiga, Naoko Aizawa, Satoru Tsuda, Yu Yokoyama, Kazuko Omodaka, Hiroshi Kunikata, Tomoki Yasui, Keiichi Kato, Hiroaki Kurashima, Etsuyo Miyamoto, Masayo Hashimoto, Toru Nakazawa

**Affiliations:** 1 Department of Ophthalmology, Tohoku University Graduate School of Medicine, Sendai, Japan; 2 Department of Ophthalmic Imaging and Information Analytics, Tohoku University Graduate School of Medicine, Sendai, Japan; 3 Department of Retinal Disease Control, Tohoku University Graduate School of Medicine, Sendai, Japan; 4 Yasui Eye Clinic, Rifu, Japan; 5 Kato Eye Center, Taiwa, Japan; 6 Japan Medical Affairs, Global R&D, Santen Pharmaceutical Co. Ltd., Osaka, Japan; 7 Department of Advanced Ophthalmic Medicine, Tohoku University Graduate School of Medicine, Sendai, Japan

**Keywords:** preperimetric glaucoma, ocular blood flow, laser speckle flowgraphy

## Abstract

**Purpose:**

To investigate the site specificity of visual field changes in eyes with normotensive preperimetric glaucoma (PPG), and to determine factors influencing visual field progression.

**Methods:**

This prospective study comprised 84 eyes of 84 normotensive PPG patients followed for at least 16 months. Optic nerve head (ONH) blood flow was assessed with tissue-area mean blur rate (MBR_T_), derived from laser speckle flowgraphy. Total deviation (TD) was measured in each sector of the Garway-Heath map to evaluate the site specificity of visual field loss. Subjects with a TD slope in the first quartile were classified as progressive, and other subjects as nonprogressive. Linear and multiple regression analyses were performed to determine factors affecting visual field progression.

**Results:**

TD in the superior sector significantly decreased in the subjects overall during the follow-up periods (−0.48 ± 1.92 dB/y, *P* = 0.025). Linear regression analysis showed that basal MBR_T_-inferior was correlated significantly with TD-superior slope (*r* = 0.332, *P* = 0.002). Furthermore, basal MBR_T_ was significantly lower in this sector in the progressive than the nonprogressive group (*P* = 0.010). Multiple linear regression analysis revealed that basal MBR_T_-inferior was the only predictive factor for TD-superior slope (β = 0.329, *P* = 0.005).

**Conclusions:**

These findings suggest that superior-sector visual field progression is most common in normotensive PPG eyes, and that reduced basal ONH blood flow is associated with visual field progression.

**Translational Relevance:**

These findings provide new insight into the involvement of ONH blood flow impairment in glaucoma pathogenesis, and demonstrate the importance of assessing ONH blood flow to determine visual field progression in normotensive PPG.

## Introduction

Preperimetric glaucoma (PPG) is defined as the presence of characteristic glaucomatous changes in the optic disc and increased vulnerability to damage in the retinal nerve fiber layer (RNFL), without the presence of visual field defects detectible with standard automated perimetry.^1,2^ Recent technologic innovations have allowed sensitive detection of glaucomatous morphologic changes and visual field impairment, even during PPG. Normally, glaucoma diagnosis is based on glaucomatous morphologic changes (detected with funduscopy) or visual field impairment (measured with automated static perimetry). Unfortunately, these methods can be difficult or misleading to correctly diagnose to PPG. Thus, new technologies, such as optical coherence tomography (OCT)-based fundus imaging and new perimetric techniques, have become available for clinical use, thereby improving the assessment of PPG progression.^3–5^ However, risk factors for PPG pathogenesis still remained poorly understood.

Recent basic research into the glaucoma field has allowed us to use technological innovations, such as laser speckle flowgraphy (LSFG) and OCT angiography, which enable the noninvasive, highly reproducible measurement of ocular blood flow. These technical innovations show that glaucoma progression may involve blood flow impairment in the optic nerve head (ONH).^6,7^ Our previous work and other investigators have suggested that the involvement of impaired ONH blood flow is not only present in glaucoma but also PPG.^8,9^ Furthermore, a long-term (5-year) retrospective study focusing on PPG outcome showed that the presence of optic disc hemorrhage and the inadequate control of intraocular pressure (IOP) were risk factors for progression.^10^ Thus, it may be necessary to consider many risk factors for the successful treatment of PPG. However, this previous long-term study was not a prospective study and did not examine ONH blood flow. Therefore, its findings have been not been confirmed in a prospective study.

The current study set out to prospectively investigate normotensive PPG eyes to determine risk factors for visual field progression, in particular the site specificity of changes in normotensive PPG eyes undergoing antiglaucoma treatment. In addition, this study assessed the involvement of systemic and ocular factors, including ONH blood flow, in visual field progression to determine risk factors likely to influence visual field impairment.

## Materials and Methods

This was a prospective, observational study. Subjects in this study were longitudinally evaluated in accordance with a protocol that included regular follow-up visits (over a 4-month interval), in which patients underwent clinical examination and several other imaging and functional tests. This research followed the tenets of the Declaration of Helsinki and was approved by the institutional review board of Tohoku University Graduate School of Medicine. Informed consent was obtained from all subjects. This study is registered at UMIN Clinical Trials Registry with the identifier UMIN000013733.

### Subjects

Subjects were enrolled at Tohoku University Hospital, Kato Eye Center, or Yasui Eye Clinic. Eligibility requirements were as follows: (1) age over 20 years, (2) open angle in gonioscopy (grade 3 or 4 in Shaffer classification), (3) refractive error within the range of +3.00 to −8.00 diopters, (4) best-corrected visual acuity 20/20 or better, (5) IOP 21 mm Hg or less in at least three examinations, (6) abnormal circumpapillary RNFL thickness (cpRNFLT) in at least one clockwise OCT scan sector, at 6, 7, 8, 10, 11, or 12 o'clock, confirmed in at least three examinations, and (7) a visual field within the normal limits of a glaucoma hemifield test, with pattern standard deviation (PSD) greater than 5%, confirmed in at least two examinations. Subjects were excluded if any of the following were present: (1) corneal abnormalities or other conditions preventing reliable applanation tonometry, (2) retinal disease affecting retinal nerve fiber layer thickness, and (3) secondary glaucoma. If both eyes of a subject met all inclusion criteria, the eye with thinner cpRNFLT was included. All subjects enrolled in this study began treatment with glaucoma medication after their diagnosis and received a topical FP agonist.

### Measurement of Clinical Parameters

All subjects underwent an ophthalmologic and general examination that comprised the following: slit-lamp and funduscopic examination, gonioscopy, IOP measurement, systemic blood pressure measurement, a visual field examination, an OCT examination, and ONH blood flow assessment. IOP was determined with Goldmann applanation tonometry under local anesthesia with a drop of 0.4% oxybuprocaine hydrochloride (Benoxil; Santen Pharmaceutical Co., Ltd, Osaka, Japan). Systolic blood pressure (SBP) and diastolic blood pressure (DBP) were measured according to the standard technique, in the brachial artery at the height of the heart with an automated monitor. Mean arterial blood pressure (MAP) and ocular perfusion (OPP) were calculated as follows: MAP = DBP + 1/3 (SBP – DBP), and OPP = 2/3 MAP − IOP. Clinical examinations were conducted at month 0 to establish basal subject background. Some subjects were previously treated with IOP-lowering agent before month 0, and these subjects underwent wash out (discontinue use of IOP-lowering medications) over 1 month to obtain basal subject background. The visual field measurement was conducted at months 4, 8, 12, and 16.

### Visual Field Evaluation and OCT Examination

**Figure 1:**
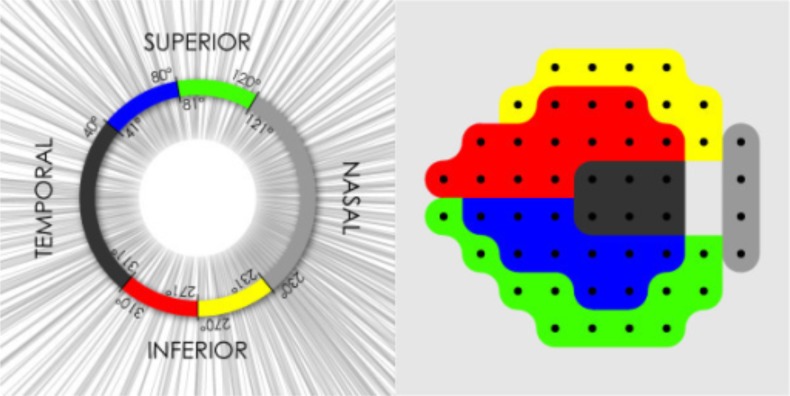
Map representing the relationship between standard automated perimetry visual field sectors and sections of the peripapillary OCT scan circle. Right panel: yellow corresponds to Superior/Nasal; red corresponds to Superior/Temporal; blue corresponds to Inferior/Temporal; and green corresponds to Inferior/Nasal. Bizios D et al. Integration and fusion of standard automated perimetry and optical coherence tomography data for improved automated glaucoma diagnostics. Bizios D, Heijl A, & Bengtsson B. Integration and fusion of standard automated perimetry and optical coherence tomography data for improved automated glaucoma diagnostics. BMC Ophthalmol. 2011;11:20. ©2011 Bizios et al.; licensee Bio Med Central Ltd. Creative Commons Attribution License (http://creativecommons.org/licenses/by/2.0)

The visual field was measured with standard automated perimetry using the Swedish Interactive Threshold Algorithm (SITA Standard 24-2) of the Humphrey Field Analyzer (Carl Zeiss Meditec Inc., Dublin, CA). Visual field measurements with fixation losses of more than 20%, or with over 33% false positives or false negatives were excluded from the analysis. Visual field function was evaluated with mean deviation (MD) and TD. The site-specific visual field was evaluated in the superior and inferior regions with the visual field sector map established by Garway-Heath et al.,^11^ which relates visual field test points to regions of the ONH (Fig. 1). Spectral-domain OCT (Cirrus HD-OCT; Carl Zeiss Meditec Inc.) was used for the evaluation of cpRNFLT. Images with signal strength less than six were considered of poor quality and were excluded from the data analysis. All OCT data were masked and reviewed at a reading center organized by experienced physicians of Tohoku University to evaluate scan errors, such as segmentation errors, blink artifacts, and out of register artifacts. The global RNFL average, RNFL thickness in the four quadrants, and RNFL thickness in the 12-o'clock scan sectors were used in the evaluation. RNFL defects were assessed clockwise in the right eyes and counterclockwise in the left eyes. The OCT software automatically classified all RFNL values as within normal limits or as abnormal (defined as results above the 95th percentile in age-matched healthy eyes).

### ONH Blood Flow Assessment with LSFG

The measurement of ocular blood flow in the ONH used LSFG-NAVI (Softcare Co. Ltd., Fukutsu, Japan), which has been approved by the Pharmaceuticals and Medical Devices Agency in Japan. The detailed principles of LSFG have been described in previous reports.^12^ Mean blur rate (MBR), an index of relative blood flow velocity, was used for the evaluation of ocular circulation. The LSFG software automatically divides the large vessel and capillary tissue areas. In this study, MBR in the capillary tissue area (MBR_T_) was used, because of its reported usefulness in intergroup comparisons.^13^ The pupils of each subject were dilated with 0.4% tropicamide (Santen Pharmaceutical Co. Ltd.) before LSFG measurement. The measurement was conducted three times at each time point, and the average MBR was used for the analysis. If the data indicated that the image quality was poor, the image was excluded from the analysis. All images were masked for review at a reading center organized by experienced technicians of Softcare Co. Ltd. and physicians of Tohoku University.

### Statistical Analysis

The data are described as the mean ± standard deviation or as numerical values (i.e., percentages). The progression of visual field loss was evaluated according to the MD or TD slope (in dB/y) per eye, based on data from at least five reliable visual field tests. A linear regression analysis, comparison of progressive and nonprogressive groups, and multiple linear regression analysis were also conducted. The linear regression analysis included the calculation of Pearson's correlation coefficient. Eyes with a MD or TD slope lower than the 25th percentile were defined as the visual field progressive group, and those with a slope higher than the 25th percentile were defined as the visual field nonprogressive group. The Student's *t*-test or Fisher's exact test were used to compare groups. A multiple linear regression analysis was used to determine basal variables affecting visual field progression. All statistical analyses were performed with SAS version 9.4 (SAS Institute Inc., Cary, NC). The significance level was set at *P* < 0.05.

## Results

### Demographic and Visual Field Progression in Normotensive Preperimetric Glaucoma Eyes

This study examined and followed 94 eyes of 94 patients who began monotherapy with an FP agonist between August 2012 and December 2014. Of the initial 94 eyes, 84 eyes of 84 patients were included in the final analysis, 10 eyes of 10 patients being excluded due to a lack of data from at least five follow-up visits. Table 1 shows the basal demographic and ocular characteristics of the included eyes. Basal IOP and MD were 16.3 ± 2.4 mm Hg and −0.36 ± 1.12 dB, respectively. Visual field changes in the normotensive PPG eyes during the follow-up period are shown in Table 2. MD and TD both significantly decreased during the follow-up period (*P* = 0.039 and *P* = 0.042, respectively). The site-specific visual field evaluation, using the sector map defined by Garway-Heath et al.,^11^ showed that visual field sensitivity in the superior region (TD-superior slope) significantly decreased, while visual field sensitivity in the inferior region (TD-inferior slope) did not change. In the present study, the eyes with a TD-superior slope lower than the 25th percentile (−1.87 dB/y) were defined as progressive (*n =* 21 eyes), and the eyes with a TD-superior slope higher than the 25th percentile were defined as nonprogressive (*n =* 63 eyes). In the TD-superior progressive eyes (*n =* 21 eyes), the eyes with a TD-inferior slope lower than the 25th percentile (−1.33 dB/y) and that higher than the 25th percentile were 14 eyes (TD-superior: progress TD-inferior: progress) and 7 eyes (TD-superior: progress TD-inferior: nonprogress), respectively.

**Table 1:**
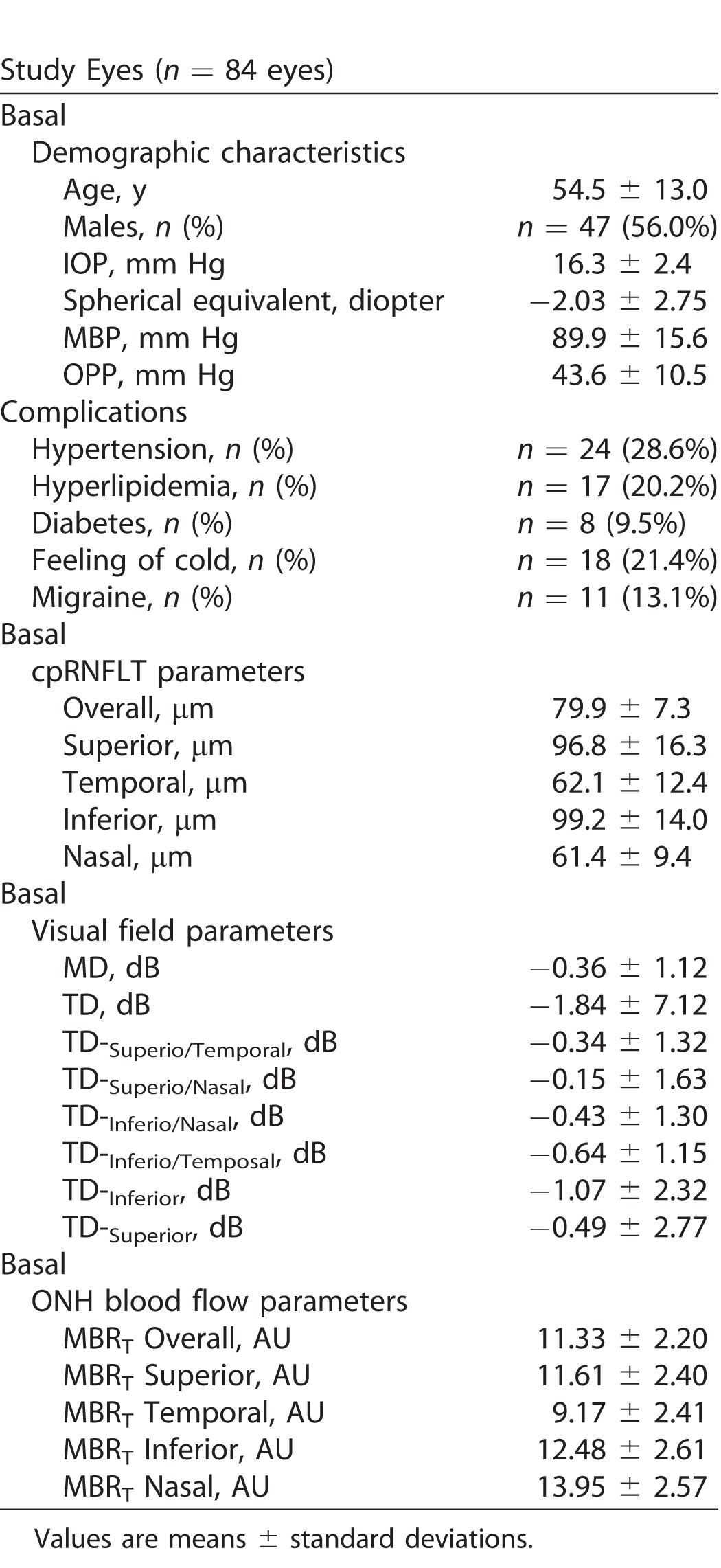
Basal Demographic/Ocular Characteristic

**Table 2:**
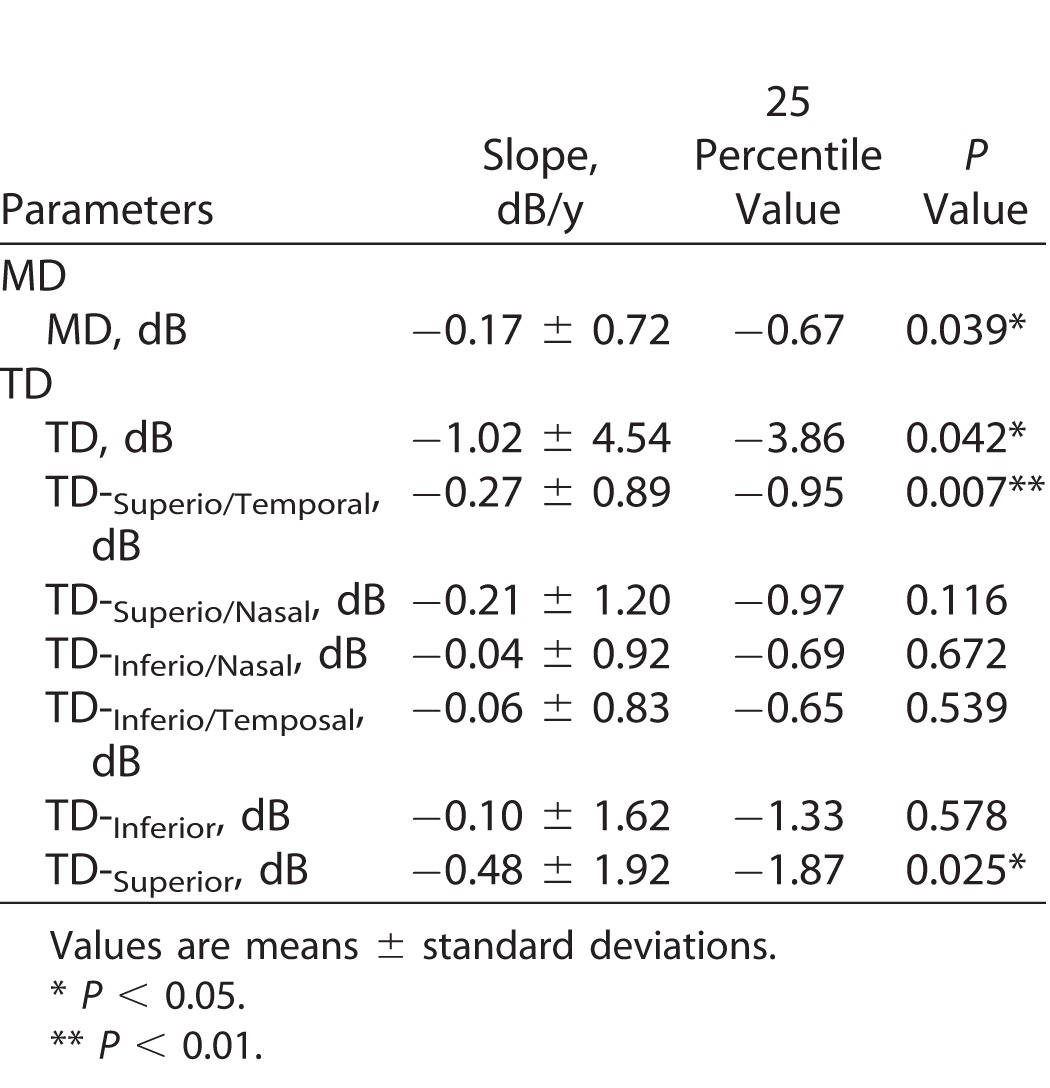
Visual Field Progression in Normotensive Preprimetric Glaucoma Eye

### Relationship Between Basal ONH Blood Flow and Visual Field Progression

**Figure 2:**
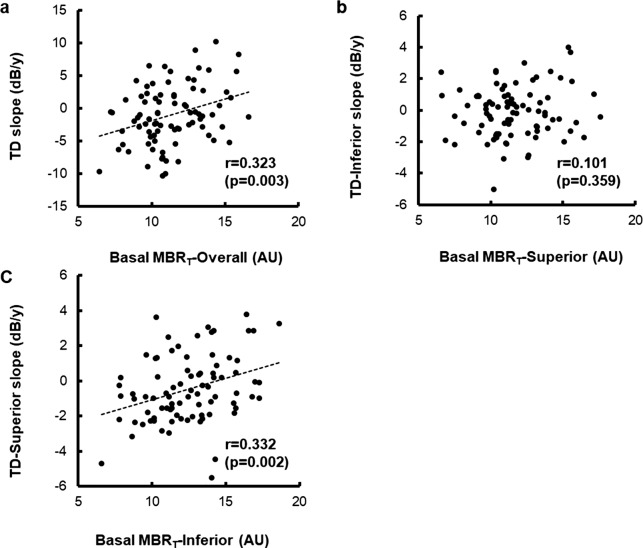
Relationship between visual field progression and basal ocular blood flow at optic nerve head. (a) The relationship between basal MBR_T_-overall and TD-slope. (b) The relationship between basal MBR_T_-superior and TD-inferior slope. (c) The relationship between basal MBR_T_-inferior and TD-superior slope.

Figure 2 shows a scatter plot of visual field progression during the follow-up period, compared with basal ONH blood flow. The linear regression analysis showed significant relationships between TD-slope and MBR_T_-overall at basal (*r* = 0.323, *P* = 0.003) and between TD-superior slope and MBR_T_-inferior at basal (*r* = 0.332, *P* = 0.002). On the other hand, no significant relationship was observed between TD-inferior and basal MBR_T_-superior (*r* = 0.101, *P* = 0.359).

### Comparison Between Progressive and Nonprogressive Eyes

The present study focused on the superior visual field, because statistically significant visual field progression was observed in this region (Table 2), and there was a significant relationship between TD slope and basal ONH blood flow in the inferior region in the site-specific visual field evaluation (Fig. 2). Table 3 shows a comparison of the visual field progressive eyes and nonprogressive eyes. The eyes with a TD slope lower than the 25th percentile were defined as progressive (*n =* 21 eyes), and the eyes with a TD slope higher than the 25th percentile were defined as nonprogressive (*n =* 63 eyes). The TD-superior slope during the follow-up period was −2.69 ± 0.99 dB/y in the progressive eyes and 0.26 ± 1.55 dB/y in the nonprogressive eyes, a significant difference (*P* < 0.001). Basal IOP in the progressive and nonprogressive eyes was 16.4 ± 2.6 and 16.2 ± 2.3 mm Hg, respectively, not a significant difference (*P* = 0.714). All relevant parameters relating to visual field sensitivity (MD and TD) at basal and cpRNFLT (overall and in the 4 quadrants) at basal were not significantly different between the groups (*P* ranged from 0.108–0.810). On the other hand, all ONH blood flow parameters (overall and in the 4 quadrants) at basal were significantly lower in the progressive eyes than in the nonprogressive eyes (*P* < 0.05).

**Table 3:**
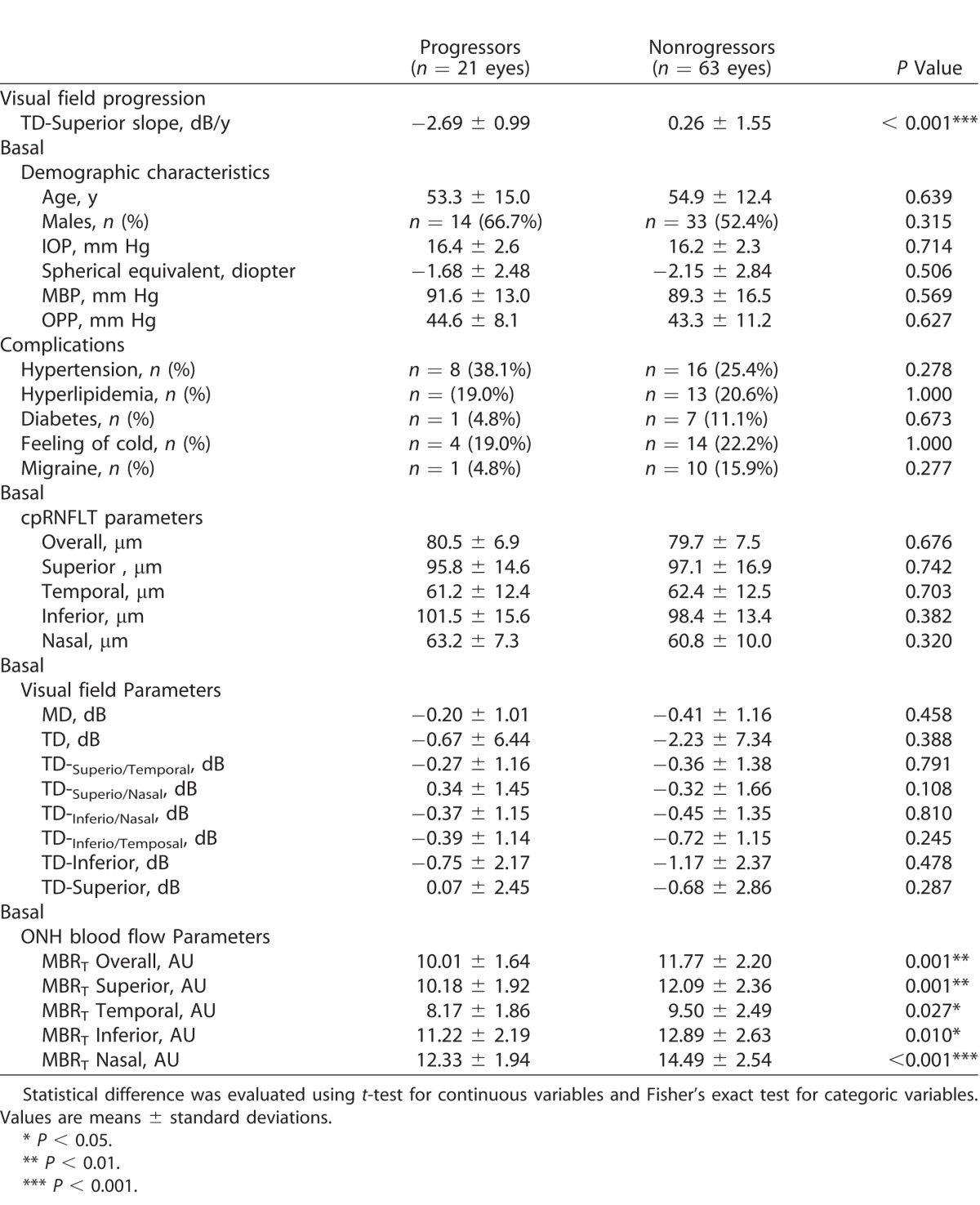
Comparison of Basal Ocular Demographic/Ocular Characteristics Between Progressors and Nonprogressors at Superior Visual Field

### Multiple Linear Regression Analysis of Visual Field Progression in the Superior Region

The contribution of various basal demographic and ocular factors to visual field progression was evaluated with a multiple linear regression analysis. Before this analysis, the multicollinearity of the variables was confirmed. This showed that there were strong correlations between age and spherical equivalent (*r* = 0.544, *P* < 0.001) and between MBP and OPP (*r* = 0.971, *P* < 0.001). Therefore, spherical equivalent and MBP were excluded from the candidate variables in the multiple linear regression analysis. Table 4 shows the results of the analysis, in which the independent basal variables were age, IOP, OPP, cpRNFLT-inferior, and MBR_T_-inferior. Basal MBR_T_-inferior was the only significant factor affecting TD-superior slope (β = 0.329, 95% confidence interval 0.046–0.243, *P* = 0.005). On the other hand, basal age, IOP, OPP, and cpRNFLT-inferior were not significant contributors to TD-superior slope (*P* ranged 0.050–0.969).

**Table 4:**
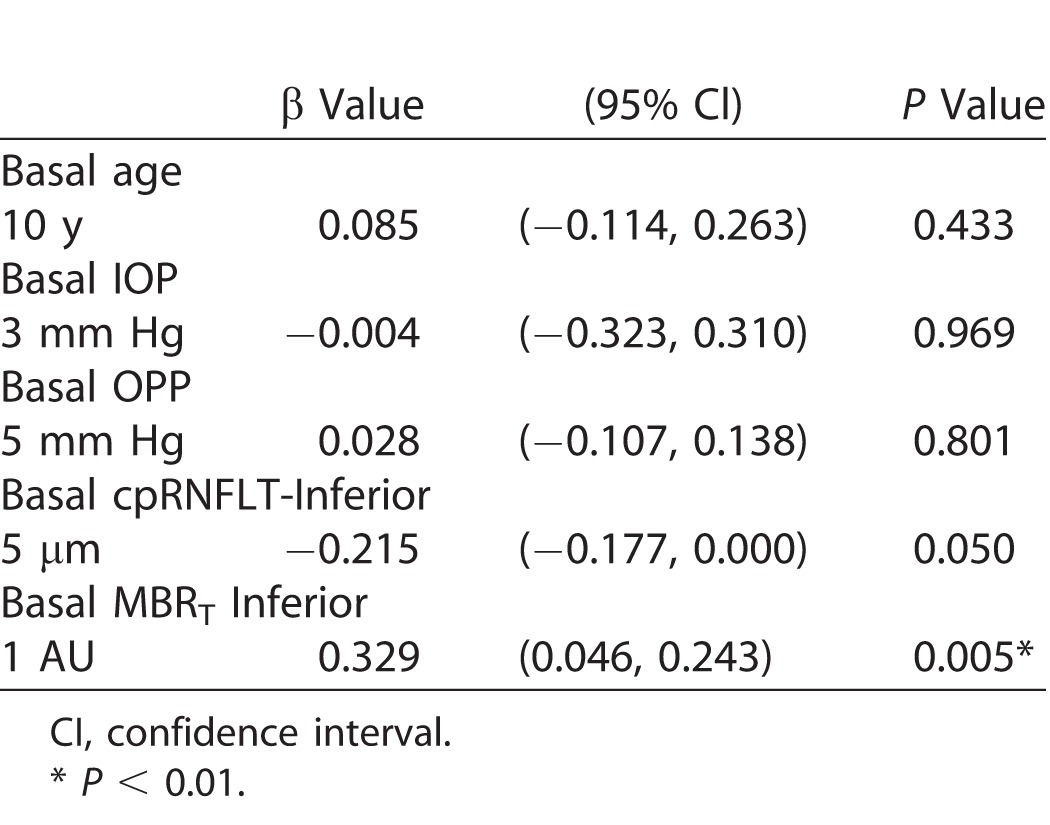
Multiple Linear Regression Analysis for Superior Visual Field Progression in Normotensive Preperimetric Glaucoma Eye

## Discussion

We conducted this prospective study to determine the site specificity of visual field changes in normotensive PPG patients undergoing IOP-lowering treatment, and to identify factors influencing visual field progression. Glaucoma progresses along a continuum that starts with the asymptomatic, preperimetric stage and ends with functional impairment in the perimetric stage.^1,2^ Here, we found that the slope of average MD (−0.17 ± 0.72 dB/y) and TD (−1.02 ± 4.54 dB/y) significantly decreased in our normotensive PPG patients during the follow-up period of this study, regardless of the IOP-lowering treatment. Previous retrospective studies have reported that the average MD slope in PPG eyes ranged from −0.09 to −0.39 dB/y, that more than 50% of eyes developed glaucomatous visual field loss within a 5-year follow-up period,^14,15^ and that IOP-lowering therapy was effective for controlling visual field progression in PPG eyes.^10^ The risk of visual field progression in untreated PPG eyes is considered to be high, so the PPG eyes enrolled in the above-mentioned studies, as well as those in the present study, received topical treatment with IOP-lowering agents. There have not been any reports on the natural course of PPG because of ethical reasons. Thus, PPG patients with risk factors such as ONH blood impairment should consider starting at least the minimal treatment.

This study used a site-specific visual field evaluation with the sector map established by Garway-Heath et al.,^11^ and found that visual sensitivity in the superior region (TD-superior slope; −0.48 ± 1.92 dB/y) significantly decreased. ^11^ Previously, Araie et al.^16^ used standard automated perimetry with the 30-2 program to study the rate of visual field loss during a 3 year-follow up period in Japanese normal-tension glaucoma patients undergoing treatment with either topical nipradilol or timolol. Araie et al. ^16^ found that the TD slope in the superior-central subfield showed significant changes with both types of treatment (−0.34 dB/y for nipradilol, and −0.29 dB/y for timolol). Although it is not possible to directly compare these visual progression rates with our current results, due to differing study designs, we consider that the inferior region of the retina, which corresponds to the superior visual field, is the region most susceptible to progression in normotensive PPG, even with the use of topical medication.

The precise mechanism of glaucomatous visual field progression remains unclear, although two main theories have been presented: mechanical and vascular. The vascular theory of glaucoma considers glaucomatous optic neuropathy to be a consequence of insufficient blood supply, due to either increased IOP or other risk factors that act to reduce ocular blood flow.^17,18^ The present study investigated factors influencing visual field progression in normotensive PPG eyes, and to the best of our knowledge, it is the first to report that ONH blood flow impairment at basal may be a predictive factor for progression. We found that the TD-superior slope was significantly correlated with basal MBR_T_-inferior in a linear regression analysis, and that there was no significant relationship with the TD-inferior slope. Additionally, a group comparison showed that basal ONH blood flow parameters were significantly lower in progressive compared with nonprogressive eyes, regardless of age, basal IOP, refractive error, systemic conditions, visual field parameters, or cpRNFLT. Moreover, a multiple linear regression analysis showed that basal ONH blood flow parameter, MBR_T_-inferior, was the only significant factor affecting the TD-superior slope. Finally, OCT variables, including cpRNFLT-inferior, as well as other variables including age, basal IOP, and basal OPP, were not associated with the TD-superior slope. These results suggest that ONH blood flow impairment acts as a primary cause of glaucomatous progression in normotensive PPG eyes.

It has been suggested that ONH blood flow impairment causes retinal ganglion cell (RGC) death by activating the production of cytotoxic substances, such as glutamate, tumor necrosis factor (TNF)-α, endothelin-1, and nitric oxide in the astrocytes, and microglia.^19^ In addition, ONH blood flow impairment directly affects mitochondria in the RGCs, resulting in less ATP being available to the cell, thus attenuating their axonal transport.^20^ Mitochondria are recognized as central players in cell death, and it is generally known that mitochondrial damage causes cell death. There is a high density of mitochondria in the RGCs due to their lack of myelination and consequent high-energy demands.^21^ Therefore, the RGCs have higher susceptibility to mitochondrial damage than other types of neuron, and blood flow impairment in the ONH selectively causes RGC death.

The present study had limitations that included a relatively small sample size and a short follow-up period. As we described, the basal cpRNFLT observed by OCT was not associated with visual field progression in the present study. However, the detailed evaluation of ONH morphology using topographic imaging devices will be needed in future studies to clearly determine the involvement of glaucomatous structural damage in the visual field progression of PPG eyes. In addition, sensitivity for detecting early glaucomatous macular visual field defects with SITA-standard 10-2 is better than that of SITA-standard 24-2, but we did not evaluate visual field defects using SITA-standard 10-2. Therefore, it is difficult to state with certainty that all eyes in the present study were in the preperimetric glaucoma stage. Furthermore, all patients underwent therapeutic intervention with an FP agonist, and none received assessments for optic disc hemorrhage during the follow-up period. The strengths of the present study include its prospective, multicenter study design and standardized follow-up and examinations, which minimized selection bias. Future prospects for the study of ONH blood flow impairment in normotensive PPG patients include, most importantly, the determination of whether treatment to improve ocular blood flow, such as with topical prostaglandin analogues, can prevent the development and progression of glaucoma.

In conclusion, this study found that the TD slope in the superior sector was significantly reduced in normotensive PPG patients, and that reduced basal ONH blood flow was a predictive factor for visual field progression. In contrast with basal ONH blood flow, there was no relationship between basal RNFLT and visual field progression during this study, even during a follow-up period of at least 16 months. These findings provide new insight into the involvement of ONH blood flow impairment in glaucoma pathogenesis, and highlight the role of this impairment in eyes with normotensive PPG, as well as the importance of measuring ONH blood flow to assess progression.
